# Fatty acid composition and genome-wide associations of a chickpea (*Cicer arietinum* L.) diversity panel for biofortification efforts

**DOI:** 10.1038/s41598-023-41274-3

**Published:** 2023-08-27

**Authors:** Sonia Salaria, J. Lucas Boatwright, Nathan Johnson, Amod Madurapperumage, Priyanka Joshi, Pushparajah Thavarajah, George Vandemark, Dil Thavarajah

**Affiliations:** 1 Plant and Environmental Sciences, Clemson University, 113 Biosystems Research Complex, Clemson, SC 29634 USA; 2 Advanced Plant Technology, Clemson University, Clemson, SC 29634 USA; 3 Grain Legume Genetics and Physiology Research Unit, USDA-ARS, Washington State University, 303 Johnson Hall, Pullman, WA 99164 USA

**Keywords:** Genetics, Plant sciences

## Abstract

Chickpea is a nutritionally dense pulse crop with high levels of protein, carbohydrates, micronutrients and low levels of fats. Chickpea fatty acids are associated with a reduced risk of obesity, blood cholesterol, and cardiovascular diseases in humans. We measured four primary chickpea fatty acids; palmitic acid (PA), linoleic acid (LA), alpha-linolenic acid (ALA), and oleic acid (OA), which are crucial for human health and plant stress responses in a chickpea diversity panel with 256 accessions (*Kabuli* and *desi* types). A wide concentration range was found for PA (450.7–912.6 mg/100 g), LA (1605.7–3459.9 mg/100 g), ALA (416.4–864.5 mg/100 g), and OA (1035.5–1907.2 mg/100 g). The percent recommended daily allowances also varied for PA (3.3–6.8%), LA (21.4–46.1%), ALA (34.7–72%), and OA (4.3–7.9%). Weak correlations were found among fatty acids. Genome-wide association studies (GWAS) were conducted using genotyping-by-sequencing data. Five significant single nucleotide polymorphisms (SNPs) were identified for PA. Admixture population structure analysis revealed seven subpopulations based on ancestral diversity in this panel. This is the first reported study to characterize fatty acid profiles across a chickpea diversity panel and perform GWAS to detect associations between genetic markers and concentrations of selected fatty acids. These findings demonstrate biofortification of chickpea fatty acids is possible using conventional and genomic breeding techniques, to develop superior cultivars with better fatty acid profiles for improved human health and plant stress responses.

## Introduction

Fats provide significant calories and energy for human well-being and healthy living. Fatty acids have several important metabolic functions in the human body, including being stored as energy sources for use under reduced blood glucose levels, helping regulate gene expression, forming cell structures, promoting cell signaling, and helping control cholesterol levels and inflammatory responses^1^. Fatty acids are classified into saturated fatty acids (SFAs), monounsaturated fatty acids (MUFAs), and polyunsaturated fatty acids (PUFAs). Meat, seed oils, seafood, and legumes contain SFAs and MUFAs ranging from 1.5 to 52 g/100 g and 0.9 to 85 g/100 g, respectively^2^, while PUFAs vary from 0.027 to 7 g/100 g. Oleic acid is a MUFA that has anti-inflammatory properties^3^ PUFAs are considered essential fatty acids (EFAs) and have two subfamilies, namely omega-6 (ω-6) and omega-3 (ω-3) fatty acids. Linoleic acid (LA; ω-6) and alpha-linolenic acid (ALA; ω-3) are important PUFAs for human health ^1,4^ . These essential fatty acids must be obtained through the daily diet^5^.

Pulse crops, including chickpea (*Cicer arietinum* L.), provide daily requirements of EFAs^6^. Chickpea originated in southeastern Turkey and is widely consumed across the world, especially in Southwest Asia^7^. Chickpea is comprised of 50–58% carbohydrates, 18–22% protein, 3.8–10% fat, and < 1% micronutrients^8^. In addition, chickpea is a rich source of prebiotic carbohydrates^9^. A human nutrition study reported that a diet rich in legumes, including chickpea, reduced weight, total cholesterol, low-density lipoprotein (LDL), and high-density lipoprotein (HDL) within eight weeks^10^. Fatty acids in chickpea also play a crucial role in plant stress responses, adaptability, and survival^11^. The degree of saturation of fatty acids in chickpea maintains membrane structure, function, integrity, fluidity, and permeability by altering their physical and physiological properties ^12,13^. Fatty acid desaturases (FADs) regulate desaturation of fatty acids in chickpea^14^, and 39 FAD genes have been identified in response to drought, salinity, and cold stress^15^. Thus, the broad range of fatty acids, suggesting wide genetic variation, can be utilized not only to explore fatty acid biofortification potential but also to breed for plant stress tolerance in chickpea.

Chickpea is one of the most consumed pulses worldwide and has great potential to fight hidden hunger and nutritional disorders and deficiencies. Global chickpea breeding programs focus on yield enhancement and disease resistance breeding. However, nutritional improvement of chickpea has become a part of breeding programs in response to increasing consumer demand for chickpea-based food products, including hummus and spreads. The nutritional quality of crops can be enhanced for increased bioavailability to the consumer using biofortification approaches based on agronomy, plant breeding, and biotechnological interventions^16^. Chickpea biofortification for micronutrients has been commonly adopted using agronomic^17–19^, breeding^20–23^, and transgenic approaches^24^. Likewise, biofortification using breeding approaches has also been suggested to improve protein concentrations in chickpea^25–27^.

Biofortification using breeding approaches offers a permanent, promising, and relatively inexpensive solution to enhance nutrients. However, biofortifying chickpea for fatty acids has yet to be addressed. This study was conducted to explore fatty acid concentrations in a chickpea diversity panel to provide information to inform fatty acids biofortification in chickpea breeding programs. The vast genetic variation in the chickpea germplasm panel for primary fatty acids, viz*.,* PA, LA, ALA, and OA, is hypothesized to be sufficient to support fatty acid biofortification efforts. A chickpea germplasm panel of 256 accessions (both *desi* and *Kabuli* types) was evaluated to (1) determine the range of concentrations of four fatty acids, viz., PA, OA, LA, and ALA, (2) estimate correlations among these fatty acids, (3) study the chickpea population structure, and (4) identify SNPs associated with fatty acid concentrations in the chickpea diversity panel.

## Results

### Phenotypic analysis of fatty acids

**Figure 1:**
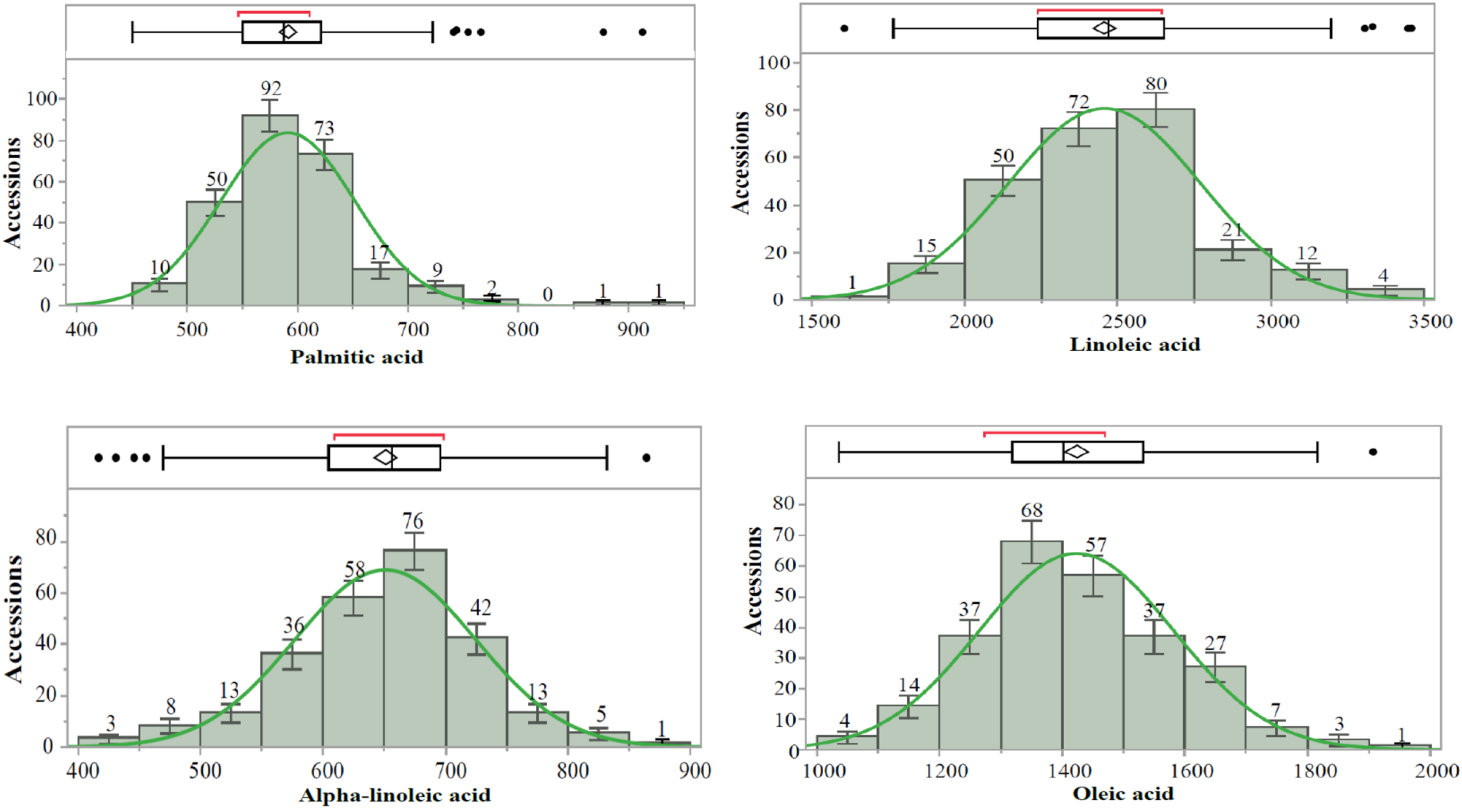
Histograms of chickpea accessions with mean values for chickpea fatty acids (mg/100 g of seeds). In the boxplots, the position of mean values is indicated by a rhombus (◊), the small line in the box represents the median, and possible outlier chickpea accessions are shown as points. The normal distribution of fatty acids data was tested using the Shapiro–Wilk test of normality and indicated by green normal curves fitted based on the mean values for chickpea fatty acids in accessions. Standard error bars are also shown.

**Figure 2:**
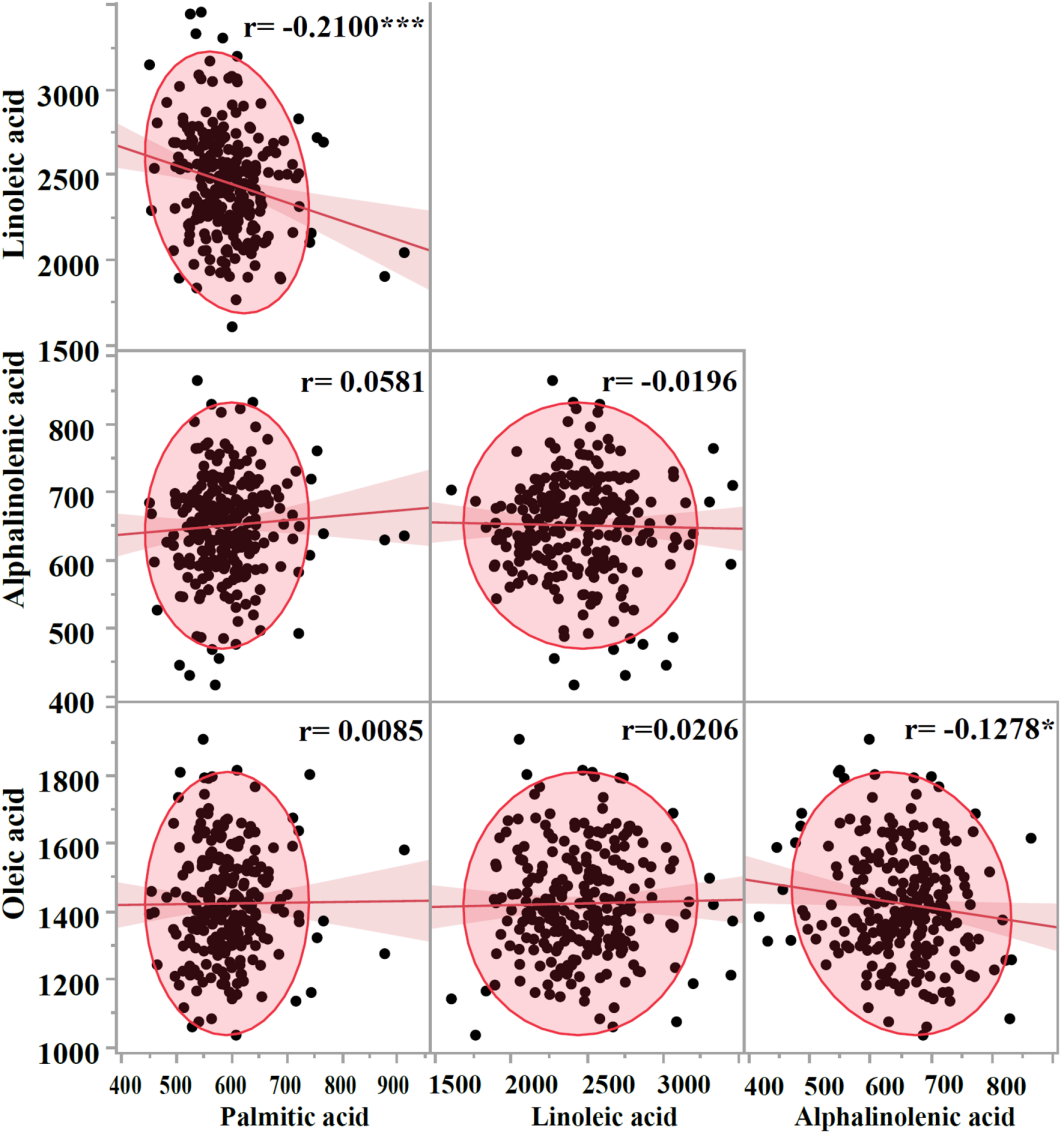
Correlation analysis of chickpea fatty acids. Significance codes: ‘***’ 0.001, ‘**’ 0.01, ‘*’ 0.05.

The chickpea diversity panel for fatty acids has accessions mainly originating in Asia and North America (77.7% and 21.5%, respectively); accessions from other continents only represent about 0.8% (Table 1). Concentrations of fatty acids, viz*.,* PA, LA, ALA, and OA, were distributed normally in the chickpea germplasm panel (Fig. 1). The mean concentrations of PA, LA, ALA, and OA were 591.13 (range 450.7–912.6) mg/100 g, 2456.39 (range 1605.7–3459.9) mg/100 g, 650.99 (range 416.4–864.5) mg/100 g, and 1423.31 (range 1035.5–1907.2) mg/g, respectively. The percent of the recommended dietary allowance (%RDA) provided by these accessions was higher for LA and ALA than for PA and OA (Table 2). Analysis of variance revealed significant genotypic effects only for PA and ALA at *p*-values < 0.01. Besides genotypic effects, all of the fatty acids varied in their replication, block, and experiment effects. The interaction effect of experiments with replication (Exp × Rep) was significant for all fatty acids. Significant interaction effects of genotype across the experiments (Exp × Geno) were only detected for PA (Table 3). Interaction effects of experiment with replication and block (Exp × Rep × Block) were significant for all fatty acids except OA. The residual mean sums of squares were high for all fatty acids. Only minor correlations were observed between concentrations of different fatty acids. PA showed a significant negative correlation with LA (r =  − 0.2100; *p*-value < 0.001). OA showed a significant negative correlation with ALA (r =  − 0.1278; *p*-value < 0.05). All other correlations were nonsignificant (Fig. 2).

**Table 1:** Origin of chickpea accessions comprising the panel for fatty acid studies.

Continent	Country	Accessions
Asia (199)	India (183)	W6 26007, W6 26008, W6 26009, W6 26010, W6 26011, W6 26012, W6 26014, W6 26015, W6 26016, W6 26017, W6 26018, W6 26022, W6 26023, W6 26024, W6 26025, W6 26026, W6 26027, W6 26028, W6 26029, W6 26030, W6 26031, W6 26032, W6 26033, W6 26034, W6 26035, W6 26036, W6 26037, W6 26038, W6 26039, W6 26040, W6 26041, W6 26042, W6 26043, W6 26044, W6 26045, W6 26046, W6 26047, W6 26048, W6 26049, W6 26050
	Iran (15)	PI 239859, PI 249981, PI 360188, PI 360223, PI 360273, PI 360279, PI 360583, PI 450953, PI 451043, PI 451187, PI 451305, PI 451431, PI 451433, PI 451453, PI 451598
	Pakistan (1)	PI 250144
Europe (1)	Spain (1)	Spanish white
North America (55)	Canada (2)	CDC Frontier, CDC Orion
	United States (53)	Billy Beans, Dwelley, Myles, Nash, Royal, Sierra, Sawyer, CA0790B0043C, CA0790B0547C, CA0890B0429C, CA13900023C, CA13900046C, CA13900049C, CA13900101G, CA13900103G, CA13900104G, CA13900119C, CA13900129C, CA13900139C, CA13900147C, CA13900151C, CA13900162C, CA15940019C, CA15940045C, CA15940049C, CA15940057C, CA15940127C, CA15940130C, CA15940141C, CA15940142W, CA15940154C, CA16940099C, CA16940136C, CA16940182C, CA16940193C, CA16940195C, CA16940200C, CA16940221C, CA16940239C, CA17900005C, CA17900011C, CA17900013C, CA17900014C, CA17900016C, CA17900017C, CA17900019C, CA17900020C, CA17900021C, CA17900028C, CA17900034C, CA17900037C, CA17900042C, CA17900046C
South America (1)	Peru (1)	PI 215702

The number of accessions from each origin are indicated in parentheses.

**Table 2:** Range, mean, and %RDA (recommended dietary allowance) of chickpea fatty acids.

Component	Range (mg/100 g)	Mean (mg/100 g)^a^	Fatty acids ^b^ (% of total fat)	%RDA ^c^
PA	450.7–912.6	591.1 ± 3.80	9.79	3.34–6.76
LA	1605.7–3459.9	2456.4 ± 19.75	40.67	21.41–46.13
ALA	416.4–864.5	650.9 ± 4.64	10.78	34.70–72.04
OA	1035.5–1907.2	1423.3 ± 9.92	23.56	4.32–7.95

^a^Mean concentration of chickpea fatty acids presented for replicates over experiments ± standard error.

^b^Fatty acids percentage is determined with total fats of 6.04 g/100 g chickpea as per 2019 reports by the USDA^42^.

^c^% RDA calculated using average recommended dietary allowance of 13.5 g/d for PA, 7.5 g/d for LA, 1.2 g/d for ALA, and 24 g/d for OA for infants and adults (males and females) according to Centers for Disease Control and Prevention survey reports^71^.

**Table 3:** Analysis of variance (ANOVA) in alpha-lattice design for chickpea fatty acids.

Source of variation	Degrees of freedom	Mean sum of squares			
		Palmitic acid (PA)	Linoleic acid (LA)	Alpha-linolenic acid (ALA)	Oleic acid (OA)
Genotypes (Geno)	254	21,765***	584413^NS^	31,997***	150274^NS^
Replication (Rep)	2	1,225,728***	10,776,035***	24930^NS^	549,931*
Block	45	83,996***	1,193,362***	126,500***	171334^NS^
Experiment (Exp)	1	32,842^NS^	75,992,635***	5269^NS^	5,419,598***
Exp × Geno	254	19,340**	470,874^NS^	20,766^NS^	162084^NS^
Exp × Rep	2	825,076***	8,625,641***	387,897***	740,900*
Exp × Rep × Block	45	46,326***	852,486*	51,049***	75,637^NS^
Residual	889	14,836	546,246	23,467	165,352

Significance codes: ‘***’ 0.001, ‘**’ 0.01, ‘*’ 0.05.

NS: Not Significant

Furthermore, prior reports for human nutrition suggest higher LA, ALA, and OA concentrations and lower PA concentrations positively impact human health^28^. In our study, the chickpea accession with the highest LA concentration was Spanish white (3459.9 mg/100 g), highest ALA concentration was W6 26,016 (864.5 mg/100 g), highest OA concentration was CA13900162C (1907.2 mg/100 g), and lowest PA concentration was W6 25894 (450.7 mg/100 g). The broad range of fatty acid concentrations in the chickpea germplasm panel (Table 2) can assist in selecting genotypes with low PA concentrations and high LA, ALA, and OA concentrations.

Principal component analysis (PCA) performed on the fatty acid data indicated principal components 1 (PC1) and 2 (PC2) explained 74.5% and 18.85% of the variance, respectively, or approximately 93% together. The PCA scatterplot showed indistinct clustering according to origin. Furthermore, the PCA biplot indicated PC1 primarily contained information for variation in LA, while PC2 primarily contained information for variation in OA (Supplementary Fig. 1). However, the first two PCs explained only minor variation in ALA and PA, which instead was explained by PC3 and PC4, respectively.

### Population structure analysis

**Figure 3:**
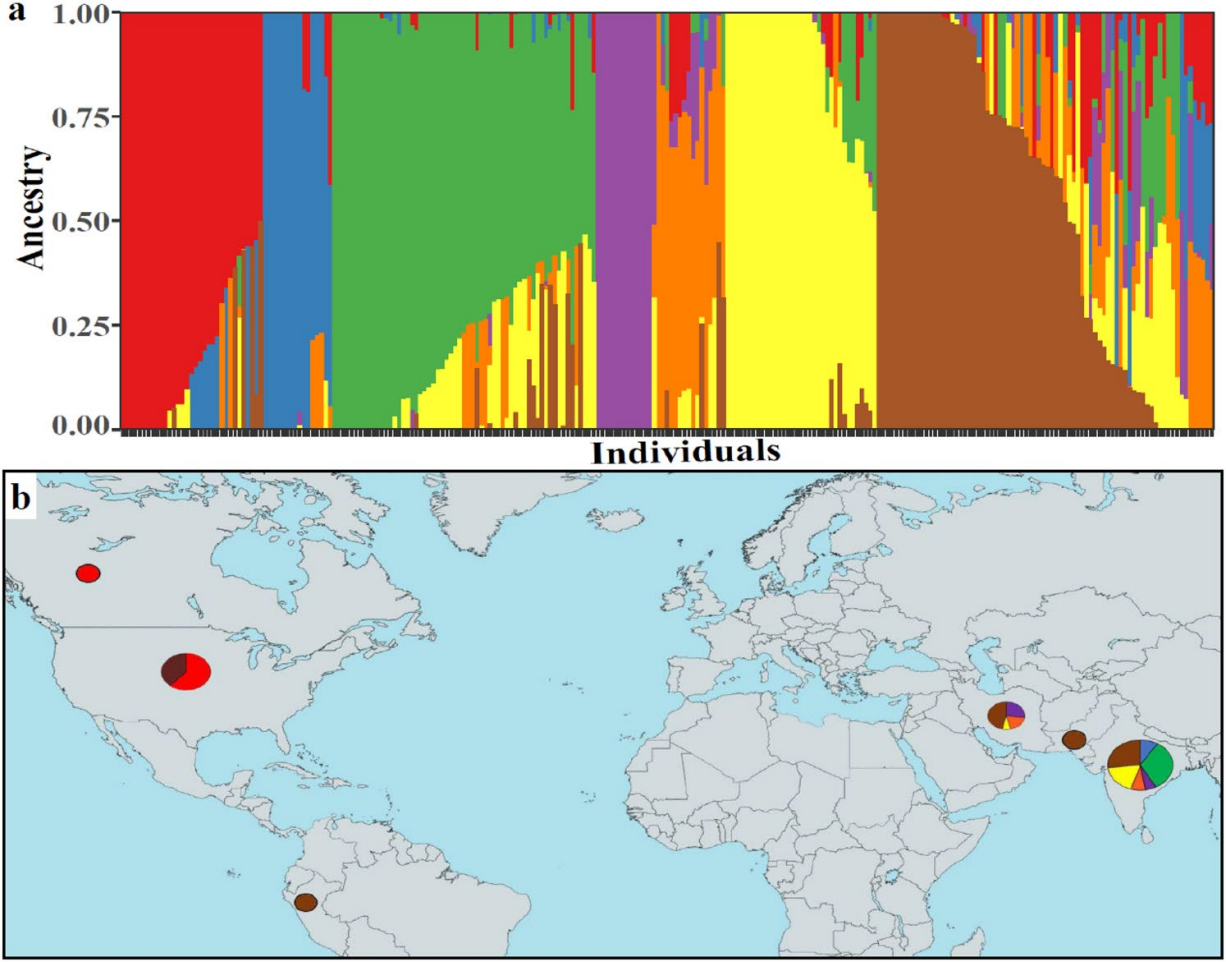
(**a**) Population admixture analysis of chickpea germplasm panel (n = 252). The individual accessions in the panel are shown along the x-axis in different colors corresponding to their ancestral estimates (y-axis) for each distinct sub-population (K = 7), and (**b**) admixture contributions of accessions from the same country of origin are indicated in pie charts whose circumference is proportional to the number of accessions. This figure was created using the R package ‘rworldmap’ (Version 1.3–6).

Population structure analysis determined seven subpopulations in the chickpea diversity panel. Subpopulation 4 (purple) had the least admixture, while subpopulation 7 (brown) had the greatest (Fig. 3a). The admixture subpopulation composition of each country of origin is shown in the map (Fig. 3b). The majority of the accessions in the chickpea germplasm panel were from India (n = 183). Consequently, most of the subpopulations were comprised either completely or partially of accessions originating from India. This is seen in subpopulations 2 (blue) and 3 (green), which exclusively include accessions of Indian origin (n = 77 combined). Similarly, of the 35 accessions in subpopulation 6 (yellow), 34 had Indian origin and one was from Iran. Sub-populations 4 (purple) and 5 (orange) comprised 23 accessions from India and seven accessions from Iran. The second highest number of accessions were from the United States (n = 52) and were found in two subpopulations: subpopulation 1 (62% of the total) and subpopulation 7 (38% of total). In subpopulation 1, almost all of the accessions originated from the United States (n = 32) except for one accession from Canada. Sub-population 7 was the most diverse ancestral group, with accessions from India (n = 49), United States (n = 20), Iran (n = 7), Peru (n = 1), and Pakistan (n = 1). Subpopulation 4 represented the smallest group, with only 14 accessions, while subpopulation 7 was the largest group, having 78 accessions.

**Figure 4:**
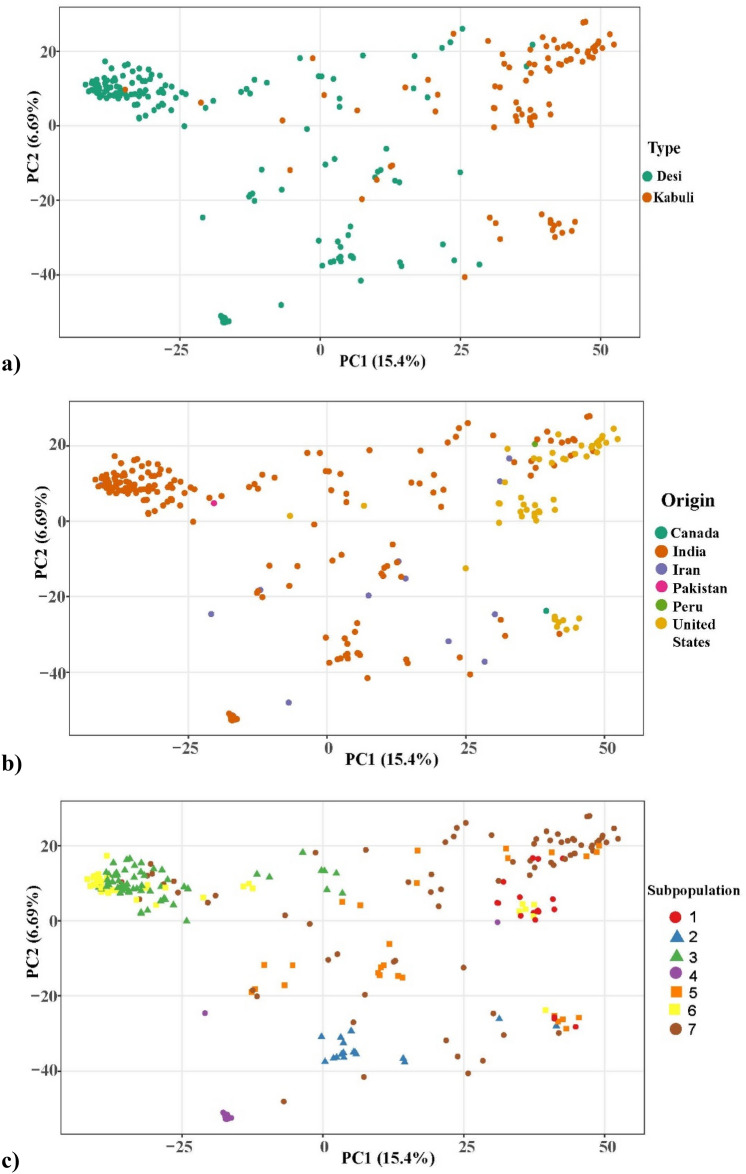
(**a**) Principal components 1 and 2 indicating accessions as points colored according to the chickpea types, (**b**) principal components 1 and 2 indicating accessions as points colored according to their country of origin, and (**c**) principal components 1 and 2 indicating accessions as points colored according to the ancestral subpopulation.

In the genetic PCA, the first two principal components (PC1 and PC2) accounted for 15.4% and 6.69% of the total variance, respectively. The *desi* and *kabuli* types form distinct clusters (Fig. 4a). PCs also separated accessions based on their origin, especially from India and the United States (Fig. 4b). The admixture subpopulations 2, 3, 4, and 6 can be seen tightly clustered relative to other subpopulations (Fig. 4c). These admix subpopulations were comprised of accessions from India as indicated by the PCA scatterplot colored by origin (Fig. 4b). Accessions from subpopulations 1, 5, or 7 did not form distinct clusters (Fig. 4c), nor did accessions originating from Iran, Pakistan, or Peru (Fig. 4b).

### Genome-wide association studies

**Figure 5:**
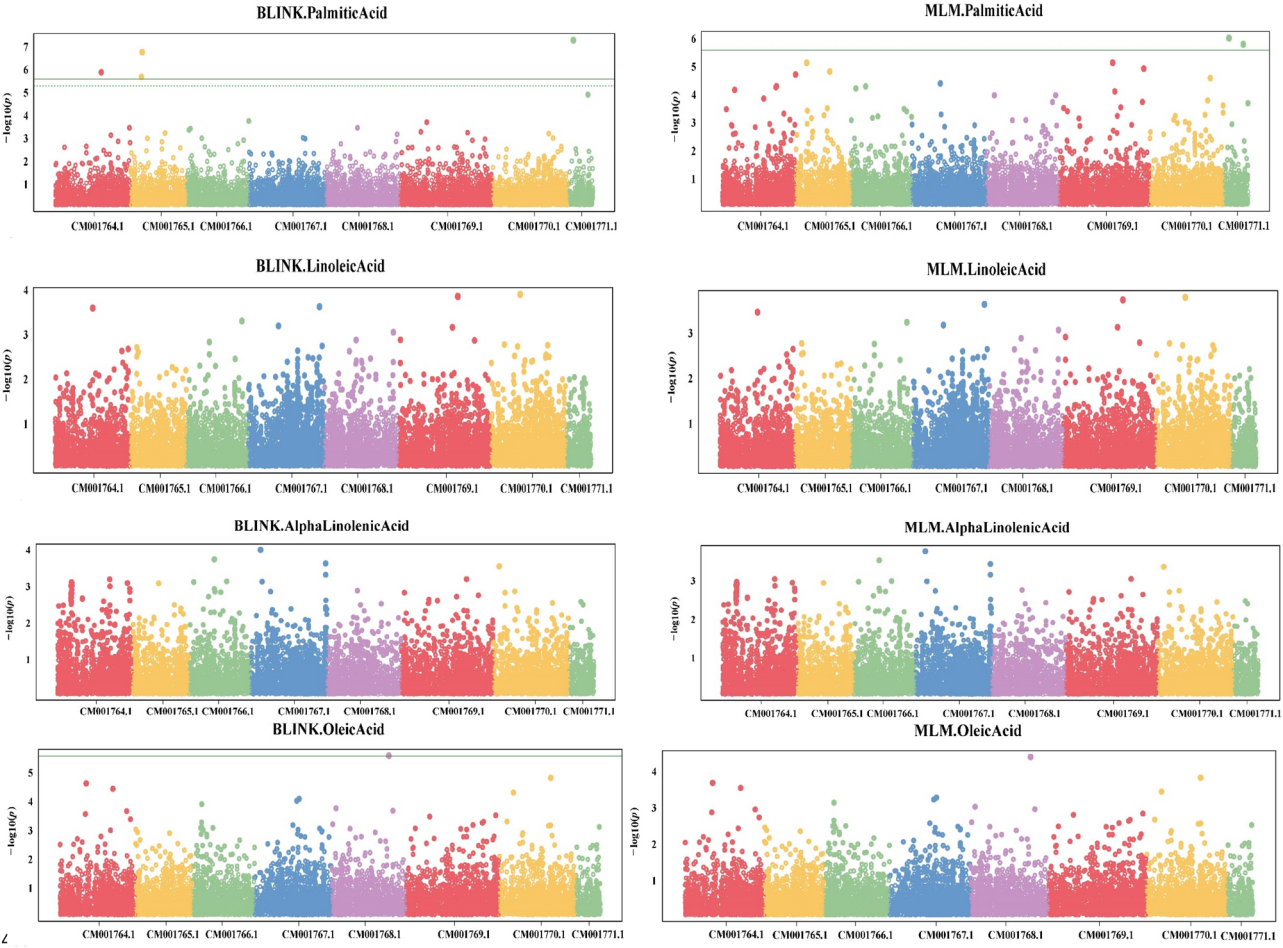
Manhattan plots from GAPIT using BLINK (Bayesian-information and Linkage-disequilibrium Iteratively Nested Keyway) and MLM (mixed linear model) for fatty acids. Significant SNPs were only found for palmitic acid with Bonferroni significance thresholds indicated by the solid green line (*p*-value < 0.05/15,927).

Of the four fatty acids examined, significantly associated SNPs were only detected for PA (Fig. 5). Five significant SNPs were found at a *p*-value < 0.05, having a minor allele frequency (MAF) ranging from 1.19 to 12.30% (Table 4). One significant SNP was identified on chromosome 1 with a MAF of 12.30%, while two were identified each on chromosome 2 (MAF: 3.37 and 4.76%) and chromosome 8 (MAF: 1.19 and 2.78%). Of these five significant SNPs, one SNP, SCM001765.1_7756123, on chromosome 2 was found within a gene (Supplementary Table 1). Also, one significant SNPs, SCM001756.1_7281701 was found with a small positive estimate for effect sizes of 0.72. Twenty-five candidate genes were identified in linkage disequilibrium (LD) blocks for variants associated with PA (Supplementary Table 1). A significant SNP, SCM001765.1_7756123 was found within a gene, *cicar.CDCFrontier.Ca_18126*, on chromosome 2.

**Table 4:** List of SNPs significantly associated with palmitic acid concentration.

SNP	Chromosome number	MAF (%)	*p*-value BLINK	*p*-value MLM
SCM001764.1_29706924	Chr 1	12.30	1.62E−06	–
SCM001765.1_7281701	Chr 2	4.76	2.58E−06	–
SCM001765.1_7756123	Chr 2	3.37	2.15E−07	–
SCM001771.1_3705203	Chr 8	1.19	6.45E−08	1.16E−06
SCM001771.1_13092034	Chr 8	2.78	–	1.93E−06

Significance threshold = 0.05/15,927 = 3.14E−06.

## Discussion

Chickpea is an important cool season legume widely consumed in diets worldwide. From an agricultural perspective, chickpea is a valuable rotation crop that improves soil health by enriching soil nitrogen pools by biological fixation, which reduces fertilizer input costs for successive crops^29^. Numerous studies have been conducted to determine the nutritional composition of chickpea protein^30–32^, carbohydrates^9,30,33–35^, micronutrients^23,31,36,37^, and fats^38–41^. However, biofortification using breeding approaches has focused mostly on protein^25–27^ and micronutrient improvements^20–23^. This is the first reported study to examine fatty acid concentrations across a chickpea diversity panel, detect SNPs significantly associated with fatty acid concentration, and identify putative candidate genes that may condition fatty acid concentrations in chickpea.

In this panel, the percent mean concentrations for PA (9.79%), ALA (10.78%), and OA (23.57%) were higher than USDA estimates for PA (8.41%), ALA (1.68%), and OA (22.62%) in chickpea^42^. However, the LA mean estimate of 40.67% was lower than the USDA estimate (43.54%). Percent concentrations of LA and OA in this panel (Table 2) were 40.67 and 23.56%, respectively, which are lower than the concentrations (57.26 and 27.98%, respectively) reported in a previous study^38^. However, a comparable PA concentration (9.79%) and much higher ALA concentration (10.78%) were found in this study than previously reported (9.69% for PA; 1.59% for ALA)^38^. Another report observed higher concentrations of LA (41.25–57.25%) and OA (27.55–42.30%), and lower concentrations of PA (8.43–9.63%) and ALA (1.68–2.68%) compared to this study^40^. A recent report on conventionally grown chickpea found lower PA (9.25%), OA (24.77%,) and ALA (1.61%) concentrations, and a higher LA (59.19%) concentration than this study^39^. This wide variation in fatty acid concentrations across different studies suggests complex inheritance of these traits and the need for robust screening of these traits across multiple environments and years. This can help to better understand the relative magnitude of genetic, environment, and their interaction effects on fatty acid concentrations in chickpea.

Chickpea fatty acid concentrations were poorly correlated, which could be attributed to a lack of genetic linkage among fatty acids or environmental influence over the genetic control of fatty acid. PA and LA were negatively correlated, which is inconsistent with a previous report in chickpea indicating a positive association^43^. However, the low correlation suggests the potential to select accessions with high overall fatty acid quality, i.e., high LA and low PA concentrations. Conversely, the significant negative correlation between OA and ALA (Fig. 2) suggests selecting a higher concentration of either fatty acid may lead to a reduced concentration of the other. This negative association between OA and ALA is consistent with prior findings^43^. A nonsignificant correlation was observed between LA and OA, and ALA, suggesting selection for higher LA concentrations will not impact OA and ALA concentrations. However, the nonsignificant correlation between LA and ALA contradicts the previous finding^44^. Correlations between fatty acids were inconsistent with the findings in soybean ^45^ and peanut ^46,47^except for PA and LA, PA and ALA, ALA, and OA in soybean ^45^. The poor correlations may be attributed to pleiotropic genetic control or loose genetic linkage among these fatty acids. The significant environmental effects can also substantially influence their genetic control and concentrations, indicating the need for further studies to confirm fatty acid correlations. In the phenotypic PCA, accessions did not form distinct clusters based on country of origin when the first two principal components were plotted (Supplementary Fig. 1). The first two components explained approximately 93% of the total variation.

Numerous GWAS have been conducted in chickpea to identify SNPs significantly associated with agronomic traits^48–51^, stress tolerance^52,53^, and nutritional traits^20,50,54,55^. However, no GWAS have been reported for fatty acids in chickpea to date. Development of genomic resources in chickpea has increased since the release of the reference genome by the International Crops Research Institute for the Semi-arid Tropics (ICRISAT), India^56^ . Recently, breeding programs have begun focusing on biofortification due to the increasing demand for quality products in the markets^57^. The GWAS analysis of chickpea fatty acids found five significant SNPs associated with PA concentration (Table 4, Fig. 5).

PA, LA, ALA, and OA levels in plants control membrane changes due to biotic and abiotic stresses^11^. In response to cold stress, high concentrations of LA increase the cold tolerance of potato (*Solanum tuberosum*)^58^, while high ALA and OA impart tolerance to drought, salinity, and cold stress in chickpea^15^. These fatty acids ultimately regulate plant health under stress conditions by membrane structural and functional maintenance, regulating integral protein functioning and preventing ion leakage^11^. The gene *cicar.CDCFrontier.Ca_20107* was associated with significant SNP SCM001771.1_13092034 on chromosome 8 (Supplementary Table 1). This gene codes for a chloramphenicol acetyltransferase-like domain, which is linked to the synthesis pathways of palmitate (esters of palmitic acid) in eukaryotes^59^ . This gene is also associated with mitochondrial fatty acid biosynthesis initiation in *Arabidopsis thaliana*^60^ and *Pisum sativum*^61^ and lipoxygenase/hydroperoxidase lyase pathways in *Oryza sativa*^62^. Some genes associated with significant SNPs were linked to lipid metabolism in plants. For example, the gene *cicar.CDCFrontier.Ca_19124*, associated with SNP SCM001769.1_7281701, was identified on chromosome 2 (Supplementary Table 1). This gene codes for a glycosyl hydrolase superfamily responsible for synthesizing sphingolipids^63^ in *Arabidopsis thaliana*. Similarly, the gene *cicar.CDCFrontier.Ca_02207,* on chromosome 8, associated with SNP SCM001768.1_3705203. This gene codes for peroxidase superfamily proteins that support peroxidase activity and response to oxidative stress in *Arabidopsis thaliana* ^64^, *Pisum sativum* ^65^ and *Nicotiana tabacum* ^66^. Although this study only identified genomic associations for PA, future studies with increased statistical power are likely to associate additional fatty acids with genomic markers. This study demonstrates the possibility of targeting specific fatty acids for chickpea improvement using molecular markers.

Admixture analysis revealed seven ancestral subpopulations within this chickpea germplasm panel (Fig. 3a), which follows previous studies in chickpea^67^ . However, some studies reported two or three subpopulations in chickpea^20,50,54,55^. These seven subpopulations represent the diverse ancestral background of chickpea. There were also completely admixed accessions indicating the blending of alleles across multiple subpopulations. Most subpopulations had higher candidacy of accessions from the same country of origin, while a few were composed of accessions from different countries (Fig. 3b). Genotypic PCA distinguished both the chickpea types, *Desi* and *Kabuli* (Fig. 4a) and also showed a close relationship of a particular type to the country of origin (Fig. 4b). This suggests the independent evolution of *desi* and *kabuli* chickpeas based on their regions of domestication as well as the geographical isolation of both types ^68^. Genotypic PCA also indicated the relationships between countries of origins and admixture subpopulations (Fig. 4b and 4c). Further genomic studies targeting and dissecting the potential of chickpea for fatty acid improvement directed toward human health and plant stress response are required.

## Conclusions

Chickpea fatty acids are crucial for regulating human health, including controlling blood cholesterols, obesity, cardiovascular diseases, etc. Additionally, these fatty acids help plants to cope with adverse environmental conditions including cold and drought stress. Chickpea fatty acids are important breeding targets for increasing plant stress tolerance. Therefore, exploring the genetic variation in fatty acid concentrations followed by utilization of promising accessions from the chickpea germplasm panel could benefit populations with increased risk of lifestyle diseases by providing ideal concentrations of fatty acids to improve their health. These findings may also assist breeders to increase abiotic stress resilience and develop new chickpea cultivars that are adapted to new environments and changing climates.

## Methods

### Experimental material

The experimental material included a chickpea germplasm panel of 256 accessions containing both *Kabuli* and *desi* types as designated by the United States Department of Agriculture, Washington, DC, United States of America (USA). The accessions in the panel were collected from different continents and countries of origin. Most accessions were from Asia (199) followed by North America (55), Europe (1), and South America (1) (Table 1).

### Field conditions

This study complies with relevant institutional, national, and international guidelines and legislation. The appropriate permissions were taken to collect seed specimens at Dr. Vandermarks’ chickpea breeding program. All entries were planted in 2020 at the Washington State University Spillman Agronomy Farm in Pullman, WA (46.73° N, 117.18° W). A seed treatment was applied prior to planting that contained the fungicides fludioxonil (0.56 g kg^-1^, Syngenta, Greensboro, NC), mefenoxam (0.38 g kg^-1^, Syngenta, Greensboro, NC), and thiabendazole (1.87 g kg^-1^, Syngenta, Greensboro, NC), thiamethoxam (0.66 ml kg^-1^, Syngenta, Greensboro, NC) for insect control, and molybdenum (0.35 g kg^-1^).

All entries were evaluated in two replicated field experiments at different locations on Spillman Agronomy Farm. Soil was Mollisols (Palouse series, fine-silty, mixed superactive, mesic, Pachic Ultic Haploxerolls)^69^. Experiment 1 (DTST1) was planted on 04/30/2020 and Experiment 2 (DTST2) on 05/29/2020. Experiments used an α-lattice design with three replications/entry. Entries were mechanically planted as a four-row plot (1.5 m) with 25 seeds/plot and 20 cm spacing between rows. Weeds were controlled by a single post-plant/pre-emergence application of metribuzin (0.42 kg ha^-1^, Bayer Crop Science, Raleigh, NC) and linuron (1.34 kg ha^−1^, NovaSource, Phoenix, AZ). All plots for Experiment 1 were mechanically harvested during 09/23/2020 – 09/25/2020. All plots for Experiment 2 were mechanically harvested during 10/19/2020–10/21/2020. Seed from each plot was cleaned and samples sent to Clemson University for fatty acid analysis.

### Fatty acid extraction

Each ground chickpea seed sample (1 g) was weighed into a glass bottle. Then 20 mL of hexane were added to each bottle to form a mixture that was sonicated at 50 °C for 30 min in a thermostatic water bath. After sonication, the supernatants were filtered under vacuum to collect hexane extracts into glass tubes. The hexane extract obtained was mixed with 10 mL of 2 M methanolic KOH and vortexed for 30 s. The samples were rested for 1 min to allow phase separation (hexane and methanol phases). The content of the hexane phase (upper phase) was diluted 100X with hexane before analysis.

The samples were analyzed using Agilent’s 8860 gas chromatography system with a 5977 B GC-mass detector (MSD). An Agilent HPS-MS UI:US0347823H column with dimensions 30 m × 250 µm × 0.25 µm was used in this study. The oven, initially at constant temperature of 40 °C, was programmed to ramp up at 10 °C per min until it reached 320 °C, which was maintained for 5 min. The inlet temperature was maintained at 300 °C. The overall run time of the analysis was 34 min. Fatty acids observed in the chromatogram were identified through the NIST (National Institute of Standards and Technology) library built on Agilent’s Mass hunter Workstation-Qualitative Analysis for GCMS and LCMS (version 10.1). Identified peaks of each fatty acid were quantified using Agilent’s Mass hunter Workstation-Quantitative Analysis for gas chromatography/mass spectrometry (GCMS) and liquid chromatography/mass spectrometry (LCMS) (version 10.1) under automatic integration.

### Phenotypic data analysis

JMP Pro 16.2.0^70^ was used to estimate fatty acid means, ranges, and correlation coefficients; generate frequency distributions; assess distribution normality; and perform PCA. Phenotypic data for all fatty acids were evaluated for outliers using the 1.5 × Inter Quartile Range rule. Outliers were removed (n = 255) before further analysis. Means and ranges were calculated by averaging across replicates and then experiments. Fatty acid distributions were visualized using JMP software to generate box plots and frequency histograms with standard error bars (Fig. 1). Histograms were fit with normal density curves. Normality was assessed using the Shapiro–Wilk test. Pearson’s correlation coefficients were estimated between fatty acids using the restricted maximum likelihood (REML) approach and were visualized on scatterplots (Fig. 2) using JMP software. Scatterplots were fit with 95% density ellipses and regression lines with 95% confidence intervals. Percent recommended dietary allowance (% RDA) was calculated for each fatty acid using average recommended dietary allowance of 13.5 g/d for PA, 7.5 g/d for LA, 1.2 g/d for ALA, and 24 g/d for OA for infants and adults (males and females) according to Centers for Disease Control and Prevention survey reports^71^ with the following formula-

% RDA = (x/average RDA for a particular fatty acid by Centers for Disease Control and Prevention survey reports) × 100

Here, x = low and high concentration of a particular fatty acids indicated by their range in Table 2.

Effects of genotype, replication, experiment, and their interaction were evaluated by conducting analysis of variance (ANOVA) for alpha-lattice design using agricolae package^72^ in R version 4.0.5. Best linear unbiased predictors (BLUPs) were estimated for each fatty acid using rstanarm package version 2.21.3 ^73^ in R according to the model: Fatty acid ~ (1|GenotypeNum) + (1|Block:Exp:Rep) + (1|Rep:Exp) + (1|Exp) + (1|GenotypeNum:Exp)"). BLUPs were used in place of means for GWAS analyses.

### Genome wide association study

**Figure 6:**
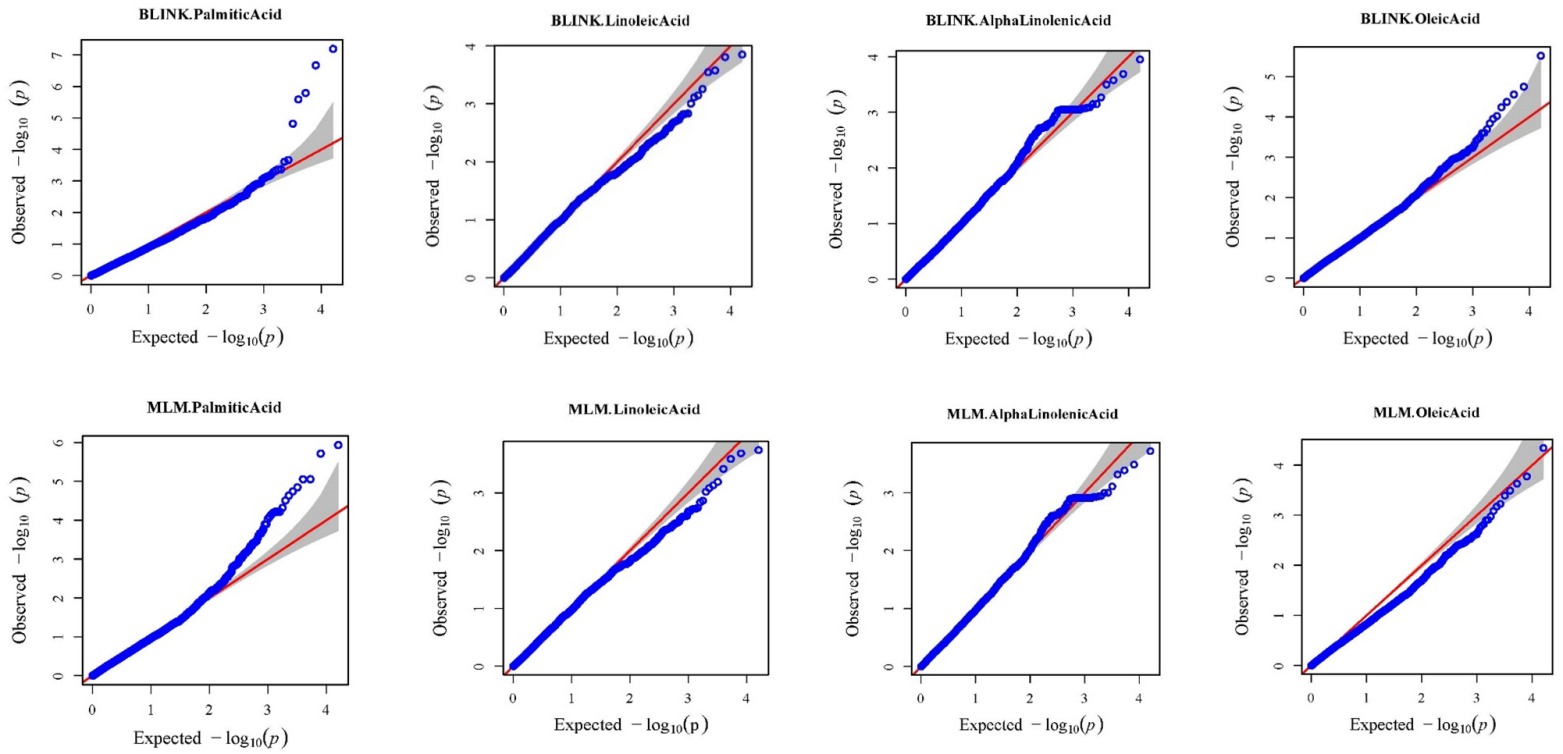
QQ plots of Palmitic acid, Linoleic acid, Alpha-linoleic acid, and Oleic acid using MLM and Blink models for genome-wide association studies from GAPIT.

Each entry was grown as single plants in a greenhouse and leaf tissue from healthy seedlings were harvested and used for SNP detection. Nucleic acid extraction from leaf tissue and detection of SNPs through the Genotyping by Sequencing (GBS) approach were performed by LGC, Biosearch Technologies (https://www.biosearchtech.com/), as previously described^51^. Processing of paired end reads (150 bp) and quality filtering was performed as previously described^51^. Filtered raw reads were aligned to the chickpea reference genome of variety CDC Frontier (*Cicer arietinum* v1.0)^74^ using the Burrow-Wheeler aligner^75^ (Li and Durbin, 2010). The Genome Analysis Toolkit^76^ (GATK; https://gatk.broadinstitute.org/) facilitated SNP calling. SNP filtering (< 20% missing data and > 5% minor allele frequency) was performed using VCFtools^77^ for quality control, resulting in 15,927 high quality SNPs. After filtering, missing genotypes were imputed using Beagle version 5.4^78^. Finally, the VCF file was converted to HapMap format in Tassel software version 5.0^79^. The fatty acid GWAS were conducted using Bayesian-information and Linkage-disequilibrium Iteratively Nested Keyway (BLINK) and Mixed Linear Model (MLM) in the Genome Association and Prediction Integrated Tool (GAPIT) version 3^80^ package in R. Manhattan plots and QQ-plots were generated during the GAPIT analysis (Figs. 5 and 6 respectively). Linkage disequilibrium (LD) was estimated using PLINK^81^ software to determine the size of LD blocks containing significant SNPs (Supplementary Table 1). Jbrowse^82^ was used to identify candidate genes in local LD with significant SNPs.

### Population structure analysis

The filtered and imputed VCF file was used to conduct both admixture analysis and PCA to evaluate population structure. Admixture in the chickpea germplasm panel was determined using ADMIXTURE version 1.3.0.^83^. The K-value with the lowest five-fold cross validation error (K = 7) was determined to be the optimal number of ancestral populations. The analysis generated a Q-matrix, which indicates the ancestral coefficients for each accession. An admixture plot was generated using the ggplot2^84^ package version 3.3.5 in R (Fig. 3a). Accessions were classified into ancestral subpopulations according to their highest ancestry coefficient (> 0.5). The rworldmap package^85^ in R was used to generate pie charts depicting the admixture composition of accessions from the same country of origin (Fig. 3b). The circumferences of the pie charts are proportional to the number of accessions sharing the country of origin. PCA was conducted using GAPIT, and the first two PCs were graphed using ggplot2^84^. PCA scatterplot points correspond to accessions and are colored according to their types (Fig. 4a), country of origin (Fig. 4b) and subpopulation (Fig. 4c).

### Supplementary Information

Supplementary Figure S1.

Supplementary Table S1.

## Data Availability

Phenotypic data for chickpea fatty acids and all the scripts used for this project are accessible at https://github.com/SSalaria5/Chickpea-fatty-acids-02-09-2023-.
