# Supplementary material for: Fatty acid composition and genome-wide associations of a chickpea (Cicer arietinum L.) diversity panel for biofortification efforts
Source: Sci Rep. 2023 Aug 27;13:14002. doi: 10.1038/s41598-023-41274-3 (PMC10460795; doi:10.1038/s41598-023-41274-3)
Supplement: Supplementary file 1 — Supplementary Figure S1. [file 41598_2023_41274_MOESM1_ESM.docx]

(a)


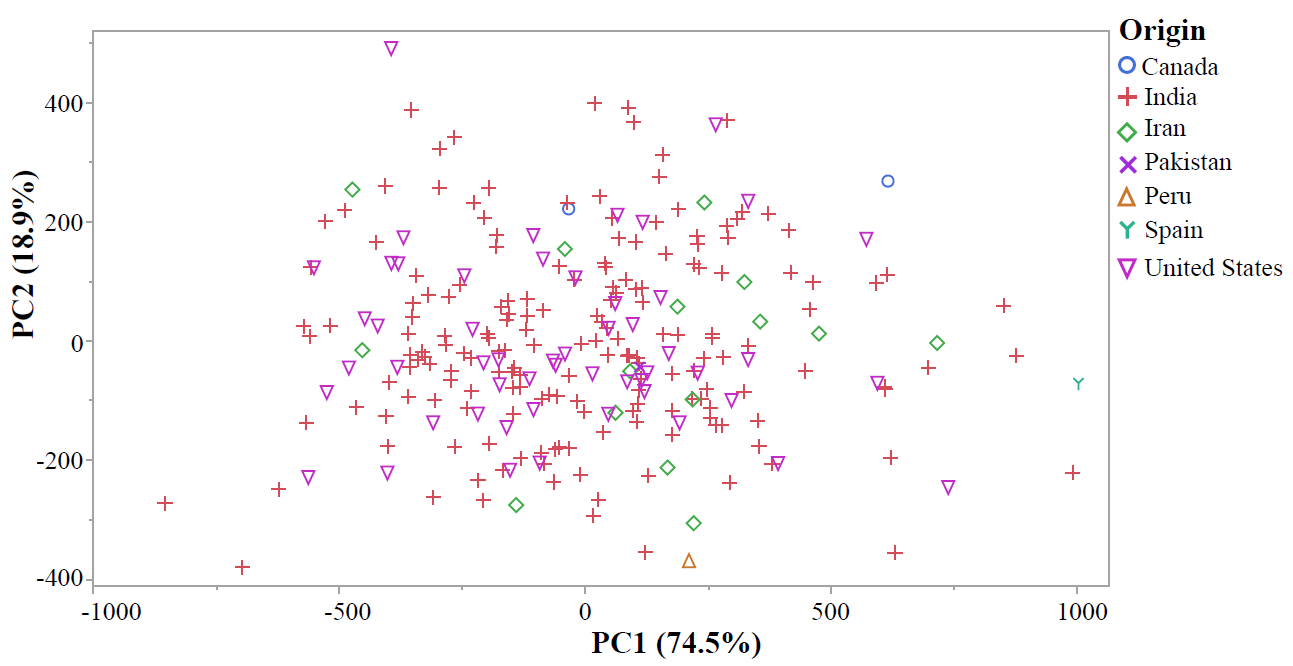


(b)

**Supplementary Figure 1:** PCA plots: (a) scatterplot showing the distribution of accessions based on their origin and (b) biplot indicating the contribution of chickpea fatty acids to principal components
